# Supplementary material for: Energy management strategy for methanol hybrid commercial vehicles based on improved dung beetle algorithm optimization
Source: PLoS One. 2025 Jan 2;20(1):e0313303. doi: 10.1371/journal.pone.0313303 (PMC11695043; doi:10.1371/journal.pone.0313303)
Supplement: Attachment — Submitted filename: Response to Reviewers.docx [file pone.0313303.s001.docx]

Li, Zhihao

1.Anhui Province Key Laboratory of Intelligent Car Wire-Controlled Chassis System, Anhui Polytechnic University, Wuhu 241000, China

2.School of Mechanical and Automotive Engineering, Anhui Polytechnic University, Wuhu 241000, China

[2220110119@stu.ahpu.edu.cn](mailto:2200120174@stu.ahpu.edu.cn)

Xiao, Ping (*Corresponding Author*)

1.Anhui Province Key Laboratory of Intelligent Car Wire-Controlled Chassis System, Anhui Polytechnic University, Wuhu 241000, China

2.School of Engineering, University of Brigeport, CT 06604, USA

[tlxp95@ahpu.edu.cn](mailto:tlxp95@ahpu.edu.cn%20)

13866376423

Pan, Jiabao

1.Anhui Province Key Laboratory of Intelligent Car Wire-Controlled Chassis System, Anhui Polytechnic University, Wuhu 241000, China

2.School of Mechanical and Automotive Engineering, Anhui Polytechnic University, Wuhu 241000, China

panjiabao@ahpu.edu.cn

Pei, Wenjun

1.Anhui Province Key Laboratory of Intelligent Car Wire-Controlled Chassis System, Anhui Polytechnic University, Wuhu 241000, China

2.School of Mechanical and Automotive Engineering, Anhui Polytechnic University, Wuhu 241000, China

[2220110151@stu.ahpu.edu.cn](mailto:2200120174@stu.ahpu.edu.cn)

Lv, Aoning

1.School of Mechanical and Automotive Engineering, Anhui Polytechnic University, Wuhu 241000, China

18110850667@163.com

Dear Editors,

Thank you very much for your supervision of the reviewing process of our manuscript with the reference number of **PONE-D-24-20196.** We also highly appreciate the reviewers’ and editors’ carefulness, conscientiousness, and the broad knowledge on the relevant research fields, since they have given us a number of beneficial suggestions. According to the reviewers’ and editors’ criticism and instruction, we have made the following revisions in terms of format and contents (which are marked in **red** or **blue** letters in manuscript):

**Journal Requirements**

**1. Please ensure that your manuscript meets PLOS ONE's style requirements, including those for file naming. The PLOS ONE style templates can be found at two PDF.**

**Response:** Thank you for your feedback. We have carefully reviewed both PDF files and made the necessary revisions throughout the manuscript as requested. For images that were modified, we indicated the changes in red font. Additionally, all images have been uploaded in TIFF format. (***Use blue markings for formatting changes.***)

2. **Please note that PLOS ONE has specific guidelines on code sharing for submissions in which author-generated code underpins the findings in the manuscript. In these cases, all author-generated code must be made available without restrictions upon publication of the work.**

**Response:** Due to proprietary improvements in the algorithm, we regret that we cannot publicly share the code related to our research. However, we are committed to transparency and reproducibility in our research. For those who wish to obtain the code, please contact us via email at (2220110119@stu.ahpu.edu.cn). We will promptly provide the code and any necessary documentation to ensure a thorough evaluation of our manuscript.

3. **We note that the grant information you provided in the ‘Funding Information’ and ‘Financial Disclosure’ sections do not match. When you resubmit, please ensure that you provide the correct grant numbers for the awards you received for your study in the ‘Funding Information’ section.**

**Response:** Thank you for pointing out our oversight and correcting our mistake. We will carefully review our funding information during the resubmission process.

4. **Thank you for stating the following financial disclosure: "The research is supported by Key Research and Development Projects in Anhui Province (2022a05020007) and the National Natural Science Foundation of China (52375227)." Please state what role the funders took in the study. If the funders had no role, please state: "The funders had no role in study design, data collection and analysis, decision to publish, or preparation of the manuscript."
   If this statement is not correct you must amend it as needed. Please include this amended Role of Funder statement in your cover letter; we will change the online submission form on your behalf.**

**Response:** Thank you for your correction. We will re-upload the cover letter and clarify the contributions made by the funders in the manuscript with the red-marked sections. We appreciate your assistance in making these changes.

The specific content is “**The research was supported by Key Research and Development Projects in Anhui Province (2022a05020007) and the National Natural Science Foundation of China (52375227). Professors Ping Xiao and Jiabao Pan made substantial contributions to the research direction design, data analysis, and manuscript revisions.”** (***In the re-uploaded cover letter, mark changes in blue font.***)

5. **Please review your reference list to ensure that it is complete and correct. If you have cited papers that have been retracted, please include the rationale for doing so in the manuscript text, or remove these references and replace them with relevant current references. Any changes to the reference list should be mentioned in the rebuttal letter that accompanies your revised manuscript. If you need to cite a retracted article, indicate the article’s retracted status in the References list and also include a citation and full reference for the retraction notice.**

**Response:** Thank you for your suggestion. We have thoroughly checked the cited references, corrected their formatting, and marked the added references in red font. We have also renumbered them accordingly in red font. If you have any further suggestions, we would greatly appreciate your feedback. (***Please see the modifications in the "References" section.***)

**Reviewer: 1**

**1.The novelty of this study is not clear. A flowchart summarizing the article should be added to the introduction section.**

**Response:** Thank you for your valuable suggestions! We take your constructive comments and make the novelty of the article clearer, the structure of the article is introduced in the last part of the introduction and a flowchart summarizing the article has been added. (***see line 113-122 and Fig 1 on page 6***)

“**The Article Flowchart is shown in Fig 1.**

**Fig 1.Article Flowchart**

2. **Authors have not provided sufficient comparison between their proposed methodology and the previous ones in the introduction. According to the current introduction, there is no contribution in the current study.**

**Response**: Thank you to the reviewers for pointing out the shortcomings in our manuscript. Based on your suggestions, we have added detailed descriptions in the introduction to highlight the differences between our proposed method and previous methods. As follows, we have listed the main innovations of our work:

(1) Compared to hybrid assemblies using conventional diesel engines, this paper uses a more environmentally friendly and economically efficient methanol engine. (***see line 97-98 on page 5***)
(2) In the traditional method, only a fuzzy control is added to regulate the charging mode and the hybrid drive mode, but the efficient operating range of the engine is not the same in these two modes, which may reduce the effect of the fuzzy control and fail to achieve the best economy. In this paper, multi-fuzzy control is added to these two modes, which can enable the engine to fully utilize the optimum economy of the vehicle in different modes of operation. We have followed reviewer’s comments and compared our work with previous approaches in more detail in the introduction. (***see line 98-101 on page 5***)
(3) To develop a more effective energy management strategy, we employ the Dung Beetle Optimization (DBO) algorithm to optimize the fuzzy controller. However, the standard DBO algorithm often falls into local optima and converges slowly. To address these issues, we propose an improved DBO algorithm that enhances solution speed and accuracy. This improved DBO algorithm is then used to derive a more efficient energy management strategy. (***see line 101-108 on page 5 and line 109-112 on page 6***)

**3.The reference numbers should be further increased. It is recommended to add the following reference.**

**Response:** We sincerely thank the reviewers for their valuable and helpful comments, and based on your suggestions, we have added a narrative on the impact of hybrid vehicles on microgrids in the second part with references to the literature recommended by you.

The specific content is “**Hybrid electric vehicles can recharge the power battery when the battery SOC falls below a set value, maintaining the balance of the battery SOC. This can reduce the dependence of hybrid vehicles on charging stations during operation and minimize the negative impact on the microgrid.**”

(***see line 130-133 on page 7 and reference 22***)

**Reviewer: 2**

1. **It is pertinent to ask the author to update the manuscript with the answer to an important question like：**

**1a) "How does the performance of the improved dung beetle algorithm compare to other optimization algorithms (such as genetic algorithms or particle swarm optimization) in terms of fuel efficiency and emissions in hybrid commercial vehicles?"**

**Response**: Thank you very much for your suggestions. Your suggestions are very constructive for this article. Based on your suggestions, we have made the following improvements.

(1) We have added a comparison of the optimization speed and accuracy between the Genetic Algorithm (GA), Particle Swarm Optimization (PSO) algorithm, Dung Beetle Optimization (DBO) algorithm, and the improved Dung Beetle Optimization (TLK-DBO) algorithm in the manuscript. (***see line 310-311 on page 17, line 320-329 on page 18, line 330-331 on page 19 and Fig 10, Table 7***).

(2) We have added the energy management strategies optimized by the GA and PSO algorithms, and included an analysis of the battery SOC value changes for these two algorithms compared with other energy management strategies. (***see line 381-383 on page 21, line 384-387 and Fig 15***)

(3) Subsequently, we included the analysis of methanol consumption for the energy management strategies optimized by the GA and PSO algorithms compared with other energy management strategies. (***see line 388-396 on page 22 and Fig 16, Table 10***)

(4) The final analysis compared the operating points of the engine under six strategies. (***see line 403-420 on page 23, line 421-426 on page 24 and Fig 17, Fig 18***)

From the comparison, it is evident that the improved Dung Beetle Algorithm achieves better global optimum economy and effectively reduces battery SOC fluctuations, thereby concentrating the engine operating points more closely within the high-efficiency zone.

**1b) How does the improved energy management strategy perform in real-world driving conditions, including various traffic scenarios and weather conditions, compared to controlled testing environments?**

**Response:** Thank you very much for your suggestion. We have included the typical operating conditions of trucks in China to further validate the effectiveness of our proposed energy management strategy. And we have added the working condition graph and the comparison graph of then consumption under this working condition.

The specific content is “**To further validate the universality and effectiveness of the proposed control strategy, we conducted tests using the China Heavy Truck Cycle (CHTC-HT) under various algorithmically optimized control strategies, as shown in Fig 19(a). The fuel consumption rates for the corresponding strategies were 144.68 L/km, 124.57 L/km, 118.98 L/km, 116.40 L/km, 115.76 L/km, and 115.07 L/km. The fuel consumption comparison results are depicted in Fig 19(b). The results verify that the improved dung beetle algorithm energy management strategy has better economy and adaptability**” (***see line 427-435 on page 24 and Fig 19***)

**(a)China Heavy Truck Cycle	(b)Fuel consumption with six strategies**

**Fig 19. China Heavy Truck Cycle and Fuel Consumption**

“ **Meanwhile, this paper further validates the feasibility and effectiveness of the optimization method through different operating conditions**.”(***see line 454-455 on page 25)***

**1c) What is the sensitivity of the improved dung beetle algorithm to different parameters such as the size of the population, number of iterations, and initial conditions?**

**Response**: We sincerely thank the reviewers for their feedback. The choice of population size, number of iterations and initial conditions in this paper is based on Ye M. and Yang G.L. who analyzed the effects of population size and number of iterations when improving the algorithm. After careful consideration, we chose a population size of 50 and 200 iterations to successfully verify the superior performance of the improved Dung Beetle Optimization (DBO) algorithm compared to Genetic Algorithm (GA), Particle Swarm Optimization (PSO), and traditional DBO algorithms.

The specific content is “**To comprehensively verify the convergence accuracy and stability of the algorithm while minimizing randomness, we conducted 50 independent runs of the GA, PSO, DBO, and TLK-DBO algorithms based on the studies of Ye M et al. [28] and Yang GL et al. [29]. Taking into careful consideration their analysis of the population size and number of iterations, we set the population size to 50 and the number of iterations to 200 for each run.**”

We appreciate the valuable feedback provided by the reviewers and hope this explanation adequately addresses your concerns. (***see line 311-315 on page 17 and reference 28,29***)

**1d) How does the improved energy management strategy affect the long-term durability and performance of the methanol engine and power battery? Are there any observed trade-offs between immediate fuel economy and long-term vehicle health?**

**Response**: Thank you very much for your meaningful feedback. In optimizing the energy management strategy, our objective was to achieve the optimal strategy that minimizes overall vehicle fuel consumption and reduces battery State of Charge (SOC) fluctuations. Ultimately, the energy management strategy we obtained improves the vehicle's overall fuel efficiency while also decreasing battery SOC variation. Reducing fluctuations in battery SOC helps extend battery life, thereby enhancing the overall lifespan of the vehicle.

The specific content is “**Comparative analysis indicates that the TLK-DBO-Fuzzy-EMS strategy maximizes the overall vehicle economy relative to the other five strategies. Based on the methanol consumption and battery SOC changes, it can be inferred that, while ensuring optimal economy, reducing SOC fluctuations throughout the entire driving cycle helps minimize instability in internal chemical reactions and occurrences of electrochemical corrosion. This contributes to extending the battery's lifespan.**” (***line 396-398 on page 22 and line 399-401 on page 23***)

2. **Can you provide more detailed explanations and justifications for the choice of Tent chaotic mapping and the specific strategies (cosine, Lévy flight, and Cauchy Gaussian mutation) used to improve the dung beetle algorithm?**

**Response:** We thank the jury experts for their corrections and apologize for the lack of explanation of our strategy for improving the algorithm. We have explained our reasons for choosing the improved strategy by revising the manuscript to describe the advantages of the improved strategy in more detail.

(1) Tent chaotic mappings are characterized by extensive randomness, continuity and smoothing, as well as enhanced diversity. Together, these properties enable the initial population to cover the entire search space uniformly, avoiding premature convergence to local optimal solutions and ensuring that the search process proceeds smoothly, balancing the needs of exploration and exploitation, thus improving the global search capability and robustness of the algorithm.Additionally, this paper combines Tent chaotic mapping with sine and cosine random assignment strategies, leveraging the advantages of chaotic and trigonometric mappings to further optimize population initialization. The sine and cosine strategies contribute to the breadth and smoothness of the search process, ensuring not only diversity within the population but also uniform distribution across the search space. (***see line 243-256 on page 14 and reference 25***)
(2) Lévy flights utilize their long-tailed distribution to effectively prevent algorithms from falling into local optima, thereby enhancing their ability to discover global optimal solutions. They can generate large step sizes, accelerating the algorithm's exploration of the entire search space and improving the efficiency of finding optimal solutions to optimization problems. This strategy is suitable for various complex and diverse optimization scenarios, showing strong adaptability without being limited by specific problem types. Additionally, Lévy flights maintain stable search performance and excel in handling complex, high-dimensional, and nonlinear problems, thereby enhancing the algorithm's robustness and practicality. (***see line 271-279 on page 15 and reference 26***)
(3) The Cauchy Gaussian mutation strategy utilizes the randomness of the Cauchy distribution to effectively enhance the algorithm's global search capability and robustness. It allows the algorithm to jump to distant positions, avoiding the risk of premature convergence to local optimal solutions, thereby enabling greater exploration and variability. This strategy enhances the algorithm's exploratory and diverse capabilities in complex, high-dimensional, and nonlinear optimization problems, significantly increasing the probability of finding global optimal solutions. Moreover, it is versatile and practical, as it is not limited by specific problem types or parameter settings. (***see line 285-296 on page 16 and reference 27***)
3. **Does the report include a comprehensive comparison between the proposed energy management strategy and existing strategies in terms of efficiency, robustness, and adaptability? If not, can this comparison be added to highlight the improvements made?**

**Response**: Thank you very much for your valuable suggestions. We have taken your advice and added a further explanation of the superiority of the energy management strategy proposed in this paper compared to rule-based and fuzzy control-based energy management strategies. The energy management strategy proposed in this paper has better adaptability and robustness compared to the rule-based strategy and can achieve optimal fuzzy control to optimize the energy management strategy.

The specific content is “**It can be seen that TLK-DBO-Fuzzy-EMS can adjust the engine's output curve based on the real-time state of the vehicle during driving, showing greater robustness and adaptability compared to Rule-EMS. Compared to Fuzzy-EMS, it finds better fuzzy control strategies to further improve vehicle economy and reduce battery SOC fluctuations.**” (***see line 414-418 on page 23***)

4. **Because of processing during production, could you please address all the in-text citation errors in the report? For instance:**

**4a) Page 11 of 40: ... specific operating conditions. Literature [13-14] selects control parameters through dynamic
4b) Page 11 of 40: ... improve the overall vehicle economy. Literature [15] utilized dynamic programming in
4c) Page 11 of 40: ... strategies. Literature [17] used a dynamic programming algorithm to obtain the global optimum
Comment: It is important to note that it is absurd and improper to cite like [1],[2],[3],[4] or [1,2,3,4]. One way to reduce erroneous in-text citations, avoid mismatching in-text citations, and properly connect them to References is to insert the author's name (i.e., Aaagah [1] or Aaagah et al. [1]). Update each reference number with the author's name following the 6th Edition of APA style.**

**Response**: Thank you for your thorough review and valuable comments on my thesis. I have noted the citation errors that you have pointed out and appreciate your attention to this matter. I have carefully reviewed the relevant chapters and confirmed that there are errors in the citations as you have stated, we apologize for this and in the manuscript we have corrected the full citations. Please feel free to let me know if you have any further suggestions or need any clarification from us. To distinguish them clearly, we marked the modified citations in red color.(***Modified all citations and marked them with red-colored font*** )

5. **The manuscript, first paragraph of the introduction lack a fact on how heat transfer affects energy shortage. Could your check a suggestion below?**

**Response:** Thank you very much for your thorough review of my paper and your valuable suggestions. I have carefully read the references you recommended. Hybrid electric vehicles can optimize energy management and thermal management systems to enhance heat transfer efficiency within the system, and the efficiency of heat transfer is critical for the utilization of energy in hybrid electric vehicles. We have incorporated citations to this literature in the revised version of our paper. Once again, I appreciate your support and assistance. Please feel free to reach out if you have any further suggestions or need clarification from my side.

The specific content is “**Hybrid electric vehicles can optimize energy management and thermal management systems to enhance heat transfer efficiency within the vehicle's energy transmission system. According to Animasaun, I.L et al. [1]the efficiency of heat transfer processes significantly impacts energy shortages by determining the effectiveness of energy utilization and conservation within a system, thereby influencing overall energy demand and supply dynamics.**” (***see line 31-35 on page 2 and reference 1***)

We have tried our best to improve the paper and made some changes in the manuscript. These changes will not influence the content and framework of the paper.

We appreciate for Editors/Reviewers’ warm work earnestly, and hope that the correction will meet with approval. We hope that these revisions are satisfactory and will be acceptable for publication in *PLOS ONE.* Once again, thank you very much for your comments and suggestions.

Wish you all the best.

Sincerely yours,

Zhihao Li, Ping Xiao, Jiabao Pan, Wenjun Pei, Aoning Lv
