# Supplementary material for: Energy management strategy for methanol hybrid commercial vehicles based on improved dung beetle algorithm optimization
Source: PLoS One. 2025 Jan 2;20(1):e0313303. doi: 10.1371/journal.pone.0313303 (PMC11695043; doi:10.1371/journal.pone.0313303)
Supplement: Attachment — Submitted filename: Response to Reviewers.docx [file pone.0313303.s002.docx]

Li, Zhihao

1.Anhui Province Key Laboratory of Intelligent Car Wire-Controlled Chassis System, Anhui Polytechnic University, Wuhu 241000, China

2.School of Mechanical and Automotive Engineering, Anhui Polytechnic University, Wuhu 241000, China

[2220110119@stu.ahpu.edu.cn](mailto:2200120174@stu.ahpu.edu.cn)

Xiao, Ping (*Corresponding Author*)

1.Anhui Province Key Laboratory of Intelligent Car Wire-Controlled Chassis System, Anhui Polytechnic University, Wuhu 241000, China

2.School of Engineering, University of Brigeport, CT 06604, USA

[tlxp95@ahpu.edu.cn](mailto:tlxp95@ahpu.edu.cn%20)

13866376423

Pan, Jiabao

1.Anhui Province Key Laboratory of Intelligent Car Wire-Controlled Chassis System, Anhui Polytechnic University, Wuhu 241000, China

2.School of Mechanical and Automotive Engineering, Anhui Polytechnic University, Wuhu 241000, China

panjiabao@ahpu.edu.cn

Pei, Wenjun

1.Anhui Province Key Laboratory of Intelligent Car Wire-Controlled Chassis System, Anhui Polytechnic University, Wuhu 241000, China

2.School of Mechanical and Automotive Engineering, Anhui Polytechnic University, Wuhu 241000, China

[2220110151@stu.ahpu.edu.cn](mailto:2200120174@stu.ahpu.edu.cn)

Lv, Aoning

1.School of Mechanical and Automotive Engineering, Anhui Polytechnic University, Wuhu 241000, China

18110850667@163.com

Dear Editors,

Thank you very much for your supervision of the reviewing process of our manuscript with the reference number of **PONE-D-24-20196.** We also highly appreciate the reviewers’ and editors’ carefulness, conscientiousness, and the broad knowledge on the relevant research fields, since they have given us a number of beneficial suggestions. According to the reviewers’ and editors’ criticism and instruction, we have made the following revisions in terms of format and contents (which are marked in **red** or **blue** letters in manuscript):

**Journal Requirements**

1. **Please review your reference list to ensure that it is complete and correct. If you have cited papers that have been retracted, please include the rationale for doing so in the manuscript text, or remove these references and replace them with relevant current references. Any changes to the reference list should be mentioned in the rebuttal letter that accompanies your revised manuscript. If you need to cite a retracted article, indicate the article’s retracted status in the References list and also include a citation and full reference for the retraction notice.**

**Response:** Thank you for your suggestion, we have thoroughly checked the references cited and made sure they are complete and correct. If you have any further suggestions, we would be grateful!

**Reviewer: 1(No Response)**

**Reviewer: 2**

**1.Start each paragraph with the definition of term or keyword it tends to announce.**

**Response:** Thank you for pointing out the shortcomings, we have revised the paragraphs starting with the missing keywords and explained them. Please contact us if you have any other suggestions.

The specific content is “**Methanol, which has a higher content of oxygen atoms, is more easily and completely combusted, resulting in lower emissions [23].**” (***see line 173-174 on page 9***)

The specific content is “**The energy management strategy for hybrid vehicles aims to keep the engine operating in its high-efficiency zone while limiting the state of charge (*SOC*) variations of the power battery, avoiding overcharging and over-discharging to extend battery life.**” (***see line 219-221 on page 12***)

The specific content is “**The input signal needs to be fuzzified before the fuzzy controller is built, and then the fuzzy output is derived from the established fuzzy rule base.**” (***see line 347-348 on page 19***)

2. **Could you please revise the introduction so that each paragraph starts with the definition of the keyword it tends to introduce?**

**Response:** Thank you for pointing out the shortcomings, we have revised the paragraph starting with the missing keyword in the introduction and explained the keyword. Please contact us if you have any other suggestions.

The specific content is “**The energy management strategy (EMS) of hybrid vehicles is the core technology, which allocates the torque distribution between the engine and the electric motor in real time during the driving process of the whole vehicle, and the goodness of the EMS affects the economy of the whole vehicle to a large extent.**” (***see line 45-48 on page 3***)

The specific content is “**The rule-based deterministic energy management strategy assigns fixed control parameters and switches the vehicle to different modes based on its current state, adhering to predetermined limits. This approach primarily relies on subjective experience to optimize economic efficiency.**” (***see line 54-56 on page 3***)

The specific content is “**The fuzzy rule-based energy management strategy fuzzyfies the precise values of inputs and uses fuzzy control rules to make decisions on the operating state of the vehicle to achieve regulation and control of the system, thereby improving the adaptability and robustness of energy management.**” (***see line 65-68 on page 4***)

The specific content is “**The energy management strategy based on global optimization is based on a cost function that has been defined and a minimum cost function for the driving conditions, resulting in the best fuel economy for the vehicle.**” (***see line 77-79 on page 4***)

The specific content is “**Energy management strategies based on transient optimization are divided into equivalent fuel minimum energy management strategies and model predictive energy management strategies. Transient optimization involves selecting the equivalent fuel consumption or total power consumption as the objective function for optimization at each moment of vehicle operation, thus determining the instantaneous optimal operating point for distributing torque between the engine and the electric motor.**” (***see line 89-94 on page 5***)

3. **Revise the introduction such that each paragraph starts with the definition of the keyword that it tends to introduce.
   Comment: There is a need to restructure the introduction section; concept definition, theoretical review, and empirical review could be easily fetched. Note that each paragraph should announce at least an important study concept. Recall that the major components of a standard paragraph under the introduction are (a) The definition of the term/concept/idea is needed to understand the exact contribution of the report to the body of knowledge. (b) The theoretical review tells us about the published aim and significant theory. (c) The empirical review tells us about published results within the scope of the subject matter.**

**Response:** Thank you for your very constructive suggestions, and as stated in revision 2, we have included definitions of keywords at the beginning of each paragraph of the introduction and explained the concept of keyword learning to make the introduction clearer and more readable.

The specific content is “**The rule-based deterministic energy management strategy assigns fixed control parameters and switches the vehicle to different modes based on its current state, adhering to predetermined limits. This approach primarily relies on subjective experience to optimize economic efficiency.**” (***see line 54-56 on page 3***)

The specific content is “**The fuzzy rule-based energy management strategy fuzzyfies the precise values of inputs and uses fuzzy control rules to make decisions on the operating state of the vehicle to achieve regulation and control of the system, thereby improving the adaptability and robustness of energy management.**” (***see line 65-68 on page 4***)

The specific content is “**The energy management strategy based on global optimization is based on a cost function that has been defined and a minimum cost function for the driving conditions, resulting in the best fuel economy for the vehicle.**” (***see line 77-79 on page 4***)

The specific content is “**Energy management strategies based on transient optimization are divided into equivalent fuel minimum energy management strategies and model predictive energy management strategies. Transient optimization involves selecting the equivalent fuel consumption or total power consumption as the objective function for optimization at each moment of vehicle operation, thus determining the instantaneous optimal operating point for distributing torque between the engine and the electric motor.**” (***see line 89-94 on page 5***)

4. **The abstract lacks clear separation between methods and results. Could you please separate the description of the methodology from the results for clarity. This helps readers understand the sequence of steps taken in the research.**

**Response:** Thank you for your suggestion, as you said we lacked a clear distinction in our abstract, as you suggested we added a clear analysis of the results in the abstract to make it easier for the reader to understand the steps of the article. If you have any other suggestions, please do not hesitate to give us your feedback!

The specific content is “**Compared to traditional rule-based energy management strategies, the optimized fuzzy control using the enhanced dung beetle algorithm continuously adjusts the torque distribution between the engine and motor based on the vehicle's real-time state, resulting in a 9.07% reduction in fuel consumption and a 3.43% decrease in battery *SOC* fluctuations.**” (***see line 22-26 on page 2***)

5. **It was written ::
    First, the rule-based energy management strategy is established by dividing the efficient working areas of methanol engine and power battery. Secondly, this paper employs Tent chaotic mapping to integrate the strategies of cosine, Lévy flight, and Cauchy Gaussian mutation, thus improving the dung beetle algorithm. This helps compensate for the traditional dung beetle algorithm's tendency to fall into local optima and enhances the algorithm's global search capability. And the fuzzy controllers for driving charging mode and hybrid driving mode are designed under the rule-based energy management strategy.**

**Could you revise or consider replacement with:**

**First, the rule-based energy management strategy is established by dividing the efficient working areas of the methanol engine and power battery. The Tent chaotic mapping is then used to integrate strategies of cosine, Lévy flight, and Cauchy Gaussian mutation, improving the dung beetle algorithm. This integration compensates for the traditional dung beetle algorithm's tendency to fall into local optima and enhances its global search capability. Subsequently, fuzzy controllers for the driving charging mode and hybrid driving mode are designed under this rule-based energy management strategy.**

**Response:** Thank you very much for pointing out our shortcomings, we think you have expressed it more clearly and accurately, and we have taken your suggestion! Thank you for your very constructive comments! (***see line 14-20 on page 1***)

6. **Line 31 - 35, it was written:
    Hybrid electric vehicles can optimize energy management and thermal management systems to enhance heat transfer efficiency within the vehicle's energy transmission system. According to Animasaun, I.L et al. [1] the efficiency of heat transfer processes significantly impacts energy shortages by determining the effectiveness of energy utilization and conservation within a system, thereby influencing overall energy demand and supply dynamics.
   Comment: Could you please revise and make it more concise? You may consider the replacement below:
    Hybrid electric vehicles (HEVs) can optimize energy management and thermal management systems to enhance heat transfer efficiency within the vehicle's energy transmission system. According to Animasaun et al. [1], the efficiency of heat transfer processes plays a crucial role in addressing energy shortages. Efficient heat transfer determines the effectiveness of energy utilization and conservation within a system, directly influencing overall energy demand and supply dynamics.**

**Response:** Thank you very much for your suggestion, we have used your formulation to make the narrative of our article clearer and more accurate! (***see line 32-37 on page 2***)

7. **Line 52, it was written: Jeoung H et al. [8] obtains the optimal logic
   Comment: Revise to: Jeoung et al. [8] obtains the optimal logic**

**Response:** Thank you for your suggestion. We have corrected these issues in the article after careful scrutiny. We appreciate your feedback; if you have any further suggestions for changes, please feel free to contact us! (***Use blue markings for changes***)

**8. Line 74, it was written: ... develop control rules. Anselma PG et al.[17] added the slope weighting method in dynamic...
Comment: Revise to:
... develop control rules. Anselma et al.[17] added the slope weighting method in dynamic...**

**Response:** As indicated in Modification Suggestion 7, we have actively revised the deficiencies you pointed out and corrected them throughout the text, thank you for your feedback! (***Use blue markings for changes***)

**9. Validation of the new results seems necessary.
Comment: The discussion of results must not stand in isolation. The authors need to compare the new results in the report with the existing related results in the literature. Consider how the results of the research article align with or differ from previous studies in the field. Discuss any similarities or contradictions and offer possible explanations for the differences. This is the best way to reduce the gap between the introduction and discussion of results.**

**Response:** Thank you for your suggestions, they make our article clearer and we discuss the results in a way that explains the differences between our approach and theirs and compares them with the energy management control strategies used in today's real-world projects. Thank you for your suggestions, and if you have any others, please feel free to contact us!

The specific content is “**Unlike most scholars who only add a single fuzzy controller in drive mode, this paper incorporates fuzzy controllers in both linear charging and hybrid drive modes, allowing for a better division of the battery SOC working area in different modes and effectively addressing the issue of excess engine output power. Compared to the currently most widely used rule-based energy management strategy, the improved DBO-optimized multi-fuzzy control in this paper reduces the overall methanol consumption of the vehicle by 9.07% and the fluctuation of the battery SOC by 3.43%, effectively enhancing the vehicle's economy, decreasing the fluctuation of the battery SOC, and greatly improving the adaptability and robustness issues of the rule-based energy management strategy.**” (***see line 461 on page 25 and see line 462-469 on page 26***)

**Reviewer: 3(No Response)**

We have tried our best to improve the paper and made some changes in the manuscript. These changes will not influence the content and framework of the paper.

We appreciate for Editors/Reviewers’ warm work earnestly, and hope that the correction will meet with approval. We hope that these revisions are satisfactory and will be acceptable for publication in *PLOS ONE.* Once again, thank you very much for your comments and suggestions.

Wish you all the best.

Sincerely yours,

Zhihao Li, Ping Xiao, Jiabao Pan, Wenjun Pei, Aoning Lv
