# Supplementary material for: Energy management strategy for methanol hybrid commercial vehicles based on improved dung beetle algorithm optimization
Source: PLoS One. 2025 Jan 2;20(1):e0313303. doi: 10.1371/journal.pone.0313303 (PMC11695043; doi:10.1371/journal.pone.0313303)
Supplement: Attachment — Submitted filename: Response to Reviewers.docx [file pone.0313303.s003.docx]

Li, Zhihao

1.Anhui Province Key Laboratory of Intelligent Car Wire-Controlled Chassis System, Anhui Polytechnic University, Wuhu 241000, China

2.School of Mechanical and Automotive Engineering, Anhui Polytechnic University, Wuhu 241000, China

[2220110119@stu.ahpu.edu.cn](mailto:2200120174@stu.ahpu.edu.cn)

Xiao, Ping (*Corresponding Author*)

1.Anhui Province Key Laboratory of Intelligent Car Wire-Controlled Chassis System, Anhui Polytechnic University, Wuhu 241000, China

2.School of Engineering, University of Brigeport, CT 06604, USA

[tlxp95@ahpu.edu.cn](mailto:tlxp95@ahpu.edu.cn%20)

13866376423

Pan, Jiabao

1.Anhui Province Key Laboratory of Intelligent Car Wire-Controlled Chassis System, Anhui Polytechnic University, Wuhu 241000, China

2.School of Mechanical and Automotive Engineering, Anhui Polytechnic University, Wuhu 241000, China

panjiabao@ahpu.edu.cn

Pei, Wenjun

1.Anhui Province Key Laboratory of Intelligent Car Wire-Controlled Chassis System, Anhui Polytechnic University, Wuhu 241000, China

2.School of Mechanical and Automotive Engineering, Anhui Polytechnic University, Wuhu 241000, China

[2220110151@stu.ahpu.edu.cn](mailto:2200120174@stu.ahpu.edu.cn)

Lv, Aoning

1.School of Mechanical and Automotive Engineering, Anhui Polytechnic University, Wuhu 241000, China

18110850667@163.com

Dear Editors,

Thank you very much for your supervision of the reviewing process of our manuscript with the reference number of **PONE-D-24-20196.** We also highly appreciate the reviewers’ and editors’ carefulness, conscientiousness, and the broad knowledge on the relevant research fields, since they have given us a number of beneficial suggestions. According to the reviewers’ and editors’ criticism and instruction, we have made the following revisions in terms of format and contents (which are marked in **red** letters in manuscript):

**Academic Editor:**

1. **In the Abstract section, SOC should be brought with the full words in the first time of using it. In addition, EMS has been defined twice in row 45 and 48, that the second one is redundant. I kindly ask you to carefully check the entire manuscript and correct such issue related to defining and using Acronyms/Abbreviations.**

**Response:** Thank you for pointing out our mistakes, through your suggestions we have revised the manuscript, perfected the problems arising from the acronyms and marked them with red font, if there are any other problems please do not hesitate to contact us. (***Use red markings for changes***)

2. **While revising your submission, please upload your figure files to the Preflight Analysis and Conversion Engine (PACE) digital diagnostic tool, https://pacev2.apexcovantage.com/. PACE helps ensure that figures meet PLOS requirements. To use PACE, you must first register as a user. Registration is free. Then, login and navigate to the UPLOAD tab, where you will find detailed instructions on how to use the tool. If you encounter any issues or have any questions when using PACE, please email PLOS at <a href="mailto:figures@plos.org">figures@plos.org. Please note that Supporting Information files do not need this step.**

**Response:** Thank you for your suggestion, we have used the tool recommended by your journal in order to make the images more in line with the journal standards and re-uploaded the diagrams modified by the tool, we hope that our operation will meet the demand. If there are any other suggestions, please contact us.

**Journal Requirements**

1. **Please review your reference list to ensure that it is complete and correct. If you have cited papers that have been retracted, please include the rationale for doing so in the manuscript text, or remove these references and replace them with relevant current references. Any changes to the reference list should be mentioned in the rebuttal letter that accompanies your revised manuscript. If you need to cite a retracted article, indicate the article’s retracted status in the References list and also include a citation and full reference for the retraction notice.**

**Response:** Thank you for pointing out our problem, we reviewed all references according to the journal's requirements, confirmed that all references were not withdrawn, and added doi numbers! We have also highlighted the revised references in red. If there are any other issues, please feel free to let us know and we would appreciate it. (***Use red markings for changes***)

We have tried our best to improve the paper and made some changes in the manuscript. These changes will not influence the content and framework of the paper.

We appreciate for Editors/Reviewers’ warm work earnestly, and hope that the correction will meet with approval. We hope that these revisions are satisfactory and will be acceptable for publication in *PLOS ONE.* Once again, thank you very much for your comments and suggestions.

Wish you all the best.

Sincerely yours,

Zhihao Li, Ping Xiao, Jiabao Pan, Wenjun Pei, Aoning Lv
